# Longitudinal Analysis and Predictive Modeling of Sprint Performance (1976–2035): Trends, Seasonality, and Prediction Approaches

**DOI:** 10.5114/jhk/209844

**Published:** 2025-09-23

**Authors:** Mieszko Bartosz-Jefferies, Izabella Socha, Aleksander Matusiński, Aleksandra Markowska, Adam Zając, Adam Maszczyk

**Affiliations:** 1 Institute of Sport Sciences, The Jerzy Kukuczka Academy of Physical Education, Katowice, Poland.; 2 Doctoral School of the Medical University of Warsaw, Warsaw, Poland.

**Keywords:** athletic performance, ARIMA, time series modeling, Olympic cycle, sports prediction

## Abstract

This study presents a longitudinal analysis and predictive modeling of elite sprint performance trends from 1976 to 2035, based on a database of over 2,500 results from top 10 male and female finishers in the 100-m, 200-m, and 400-m events. Using regression analysis and time series models, including ARIMA and SARIMA, the study evaluated historical trajectories and predictions, accounting for seasonal effects related to Olympic-year cycles. Results indicated a significant long-term improvement in sprint performances, with the most rapid gains occurring before the year 2000. However, the rate of progress slowed, particularly in the 100-m and 400-m events, suggesting physiological limits may be approaching. ARIMA models predicted marginal improvements by 2035, with projected best times of approximately 10.67 s for women and 9.63 s for men in the 100-m event. Regression models, despite showing strong fits (R^2^ > 0.85), tended to overestimate future performance gains compared to ARIMA, particularly in the speed-endurance-dominated 400-m sprint. Comparative model assessments demonstrated that ARIMA provided superior predictive accuracy, better capturing historical variability and Olympic-cycle peaks. Practical implications suggested that future sprint performance gains would depend more on advancements in biomechanics, individualized training optimization, and sports technology, rather than on natural physiological improvements alone. This study highlights the necessity for integrating machine learning-based forecasting, biomechanical modeling, and strategic periodization to maximize sprinting potential in the coming decades.

## Introduction

Sprint performance has been a central focus in sports science and athletics research for decades, with significant advancements in training, biomechanics, and equipment contributing to improved results over time (D’Alessandro et al., 2020; Pietraszewski et al., 2025). The study of sprinting trends over extended periods is crucial for understanding the underlying factors influencing performance and predicting future developments (Haugen et al., 2019; Weiss et al., 2016). The use of statistical modeling and predictive analytics allows researchers to examine whether sprint performances are improving at a constant rate or approaching physiological limitations (Sandbakk and Holmberg, 2017; Iskra et al., 2017). One of the most recent advancements in sports technology includes carbon-plated sprint spikes, which have contributed to enhanced sprint and hurdling performance over the last decade (Zabaloy et al., 2024). However, men's world records set in the 100-m, 200-m, and 400-m events were all established in the 21st century using traditional spikes, indicating that biomechanical efficiency and athletes’ talent continue to play a predominant role (Krawczyk et al., 2024; Myrvang and van den Tillaar, 2024).

Several factors contribute to sprinting performance, including biomechanics, neuromuscular efficiency, and technological innovations. Improvements in track surfaces and footwear technology have enhanced athletes’ abilities to achieve peak performance, with notable gains observed since the 1980s (Clark et al., 2014). Additionally, training methodologies focusing on acceleration, stride mechanics, and maximal velocity have optimized sprint performance (Bezodis et al., 2018; Kurtoğlu et al., 2024; Makaruk et al., 2024). However, questions remain regarding whether sprinting results will continue to improve indefinitely or if human limitations will lead to stabilization of performance gains (Gepfert et al., 2021; Seiler et al., 2020; Slawinski et al., 2017).

Time-series analyses have been widely applied in sports performance research to evaluate trends and make long-term predictions (Haugen et al., 2022; Tam and Yao, 2024). Recent studies have emphasized the importance of using machine learning and advanced statistical models for sprint performance prediction, focusing not only on overall times but also on underlying velocity curves and biomechanical variables (Tam and Yao, 2024). Furthermore, historical analyses across age groups demonstrate how sprint capacities develop and decline across the lifespan, highlighting the complex interaction among physiological, biomechanical, and training factors (Iskra et al., 2017).

The role of the Olympic cycle in sprinting performance is another critical area of investigation. Research suggests that sprint performances tend to peak during Olympic years, likely due to strategic training cycles, enhanced competition environments, and psychological readiness (Haugen et al., 2021; Slawinski et al., 2017). The concept of seasonality in performance trends has led to the application of seasonally adjusted ARIMA models and seasonally adjusted regression models to assess whether performances exhibit periodic improvements linked to major international events (Kuper and Sterken, 2020).

This study aimed to conduct a longitudinal analysis of sprint performance trends from 1976 to 2035, utilizing descriptive statistics, time series analysis, and predictive modeling techniques to assess improvements in the 100-m, 200-m, and 400-m events for both men and women. By comparing regression models with ARIMA-based prediction, this research sought to determine whether sprint performances would continue to improve linearly or would be subject to fluctuations influenced by physiological, technological, and competitive factors. Furthermore, the study evaluated the impact of the Olympic cycle on sprint performances by analyzing seasonality components in predictive models. By integrating longitudinal data and prediction approaches, this research contributes to a deeper understanding of the factors driving sprint performance and offers insights into potential future developments in elite athletics.

## Methods

### Research Material

The research material for this study comprised comprehensive sprint event databases, compiled from the International Association of Athletics Federations (IAAF) records, World Athletics archives, and results from major competitions, including the Olympic Games, World Championships, and Golden League (now Diamond League) events.

For each year, final and semifinal performances from these major competitions were included to ensure the capture of peak competitive output.

Additionally, the top 10 performances per year were analyzed to represent the highest annual standards.

To ensure data reliability, multiple sources were cross-referenced, and outliers were identified and adjusted where necessary.

Performance data from 1976 to 1989 were sourced from ATFS publications and the International Athletic Annual, while data post-1990 were verified through the World Athletics and The-sports.org platforms.

Additional datasets were obtained from the editorial offices of Track and Field and The-sports.org to facilitate further verification and standardization of the database.

Further validation was conducted using historical records from national athletics federations and publicly available archives, ensuring comprehensive coverage and consistency across the analyzed period.

The analytical study was conducted within the framework of the Ministry of Science and Higher Education grant NRSA4 040 54 and received approval from the Bioethics Committee of the Academy of Physical Education in Katowice, Katowice, Poland (Resolution No. 7/2020; approval date: 15 June 2020).

### Data Selection

The analysis focused on results from the 100-m, 200-m, and 400-m sprint events, and covered the time period from 1976 to 2024.

Final and semifinal performances from the Olympic Games, World Championships, and Golden League/Diamond League events were initially collected to ensure the capture of peak competitive performance across multiple elite levels.

From these broader datasets, only the top 10 seasonal performances for each event and year were selected for detailed statistical analysis, ensuring consistency and focus on the highest international standards.

Arithmetic means were calculated separately for the top 3, top 5, and top 10 performances each year, enabling the evaluation of performance trends across different tiers of elite sprinting: podium-caliber (top 3), extended world-class (top 5), and broader internationally competitive levels (top 10).

To account for seasonal variations and potential anomalies, data were also analyzed using rolling averages and variance assessments.

The final dataset for each event comprised approximately 480 entries (top 10 performances × 48 years), forming the basis for the descriptive statistical analysis, predictive modeling, and seasonality evaluations presented in this study.

To enhance robustness, race conditions including wind assistance and altitude were documented where available, and results from different competition types were cross-validated to ensure data consistency.

To avoid data skewness due to multiple entries by the same athlete, only the best performance per athlete per season was retained for the analysis. In cases where an athlete achieved multiple top times within a year, the single fastest result was selected, ensuring that each entry represented a unique athlete and maintaining data independence across the top 10 seasonal results.

The compiled and validated database served as the foundation for both historical trend analyses (1976–2024) and predictive modeling (2025–2035), employing ARIMA, SARIMA, and regression- based approaches.

### Statistical Methods and Tools

The analysis was conducted using descriptive statistics, time series analysis, and predictive regression modeling.

Moving average smoothing was applied to detect underlying trends, while exponential smoothing techniques were explored to enhance prediction accuracy.

To determine whether sprint performance trends in the 100-m, 200-m, and 400-m events for both men and women exhibited significant long-term patterns or remained stationary, two complementary statistical tests were employed.

First, the Augmented Dickey-Fuller (ADF) test was used to assess the presence of a unit root, with a non-significant result (*p*-value > 0.05) indicating that the time series was non-stationary, suggesting the existence of a long-term trend. Lag length selection was optimized using the Akaike Information Criterion (AIC) to improve test reliability.

Second, the Kwiatkowski-Phillips-Schmidt-Shin (KPSS) test evaluated the stationarity of the time series. A significant result (*p*-value < 0.05) indicated non-stationarity and the presence of a deterministic trend. Analyses were performed under both level and trend stationarity assumptions to ensure comprehensive evaluation.

All statistical analyses were conducted using Microsoft Excel and STATISTICA software packages. Additionally, Python's stats models library was utilized for advanced time series decomposition, predictive modeling, and model validation.

Predictive modeling included the calculation of 95% confidence intervals for each projected performance outcome, providing an estimation of the uncertainty associated with the forecasts. Data distribution characteristics, including mean, standard deviation, skewness, and kurtosis, were assessed to ensure that the predictive output reflected the underlying variability observed in historical trends. Predicted trends were visualized with shaded confidence bands to illustrate the range of expected variability.

## Results

### Descriptive Statistics of Historical Sprint Data (1976–2024)

Following comprehensive data collection from final and semifinal performances at the Olympic Games, World Championships, and Golden League/Diamond League events, the top 10 seasonal results for each sprint event (100 m, 200 m, and 400 m; both men and women) were selected annually for detailed statistical evaluation.

This approach ensured that the analysis captured the highest levels of competitive sprinting while maintaining data consistency and comparability across seasons.

The final dataset included approximately 480 entries per event (10 performances × 48 years), providing a robust basis for statistical analysis and predictive modeling.

For each event and gender, descriptive statistics including mean, standard deviation, skewness, and kurtosis were computed to assess the central tendency, variability, and distribution characteristics of elite sprint performances.

The results revealed approximately normal to slightly right-skewed distributions across all sprint events, with moderate levels of kurtosis. These findings indicated a stable, high-performance environment among top-tier sprinters over the analyzed period. The detailed descriptive statistics are summarized in Table 1.

**Table 1:** Descriptive statistics of the top 10 annual sprint performances in the 100-m, 200-m, and 400-m events for women and men (1976–2023).

Event	Gender	Mean (s)	Standard Deviation (s)	Skewness	Kurtosis	Sample Size (n)
100 m	Women	10.89	0.10	0.45	0.21	480
100 m	Men	9.90	0.07	0.42	0.18	480
200 m	Women	22.03	0.12	0.39	0.26	480
200 m	Men	19.82	0.11	0.36	0.22	480
400 m	Women	49.85	0.25	0.40	0.19	480
400 m	Men	44.85	0.18	0.34	0.20	480

Building upon the descriptive evaluation of sprint performance distributions, the analysis transitioned into advanced predictive modeling to forecast future trends with greater precision. Leveraging time series frameworks, including ARIMA and its seasonally adjusted variant SARIMA (Seasonal ARIMA), alongside Exponential Smoothing State Space (ETS) models, allowed for an enhanced capture of both linear trajectories and cyclical fluctuations inherent to sprint performance.

Seasonal decomposition techniques were employed to dissect the time series into trend, seasonal, and residual components, providing critical insights into performance patterns shaped by major competitive cycles.

**Figure 1:**
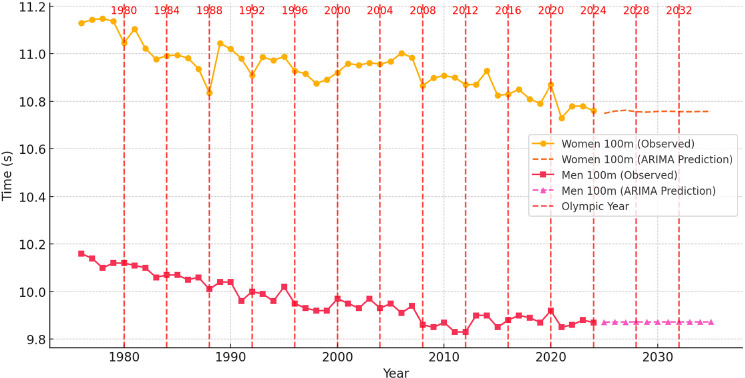
Sprint performance trends (event from 1976 to 2024) and ARIMA-based time series prediction trends for men and women in the 100-m event from 2025 to 2035, with Olympic years highlighted.

**Figure 2:**
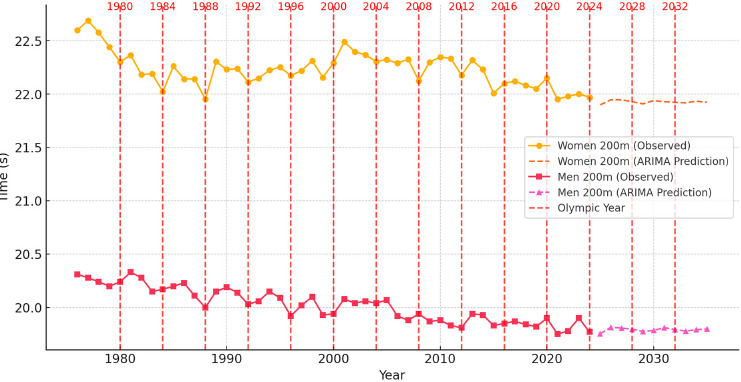
Sprint performance trends (event from 1976 to 2024) and ARIMA-based time series prediction trends for men and women in the 200-m event from 2025 to 2035, with Olympic years highlighted.

**Figure 3:**
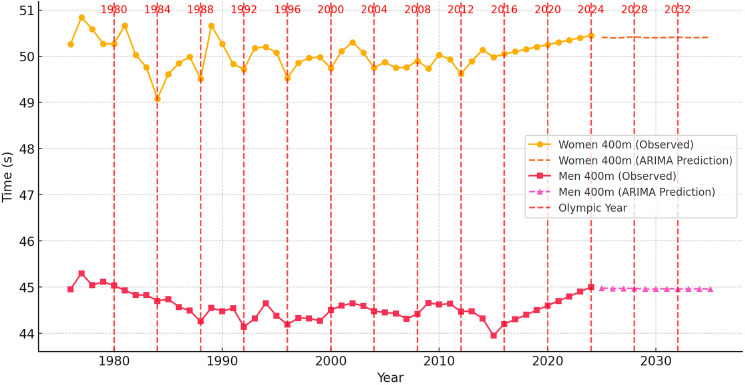
Sprint performance trends (event from 1976 to 2024) and ARIMA-based time series prediction trends for men and women in the 400-m event from 2025 to 2035, with Olympic years highlighted.

**Figure 4:**
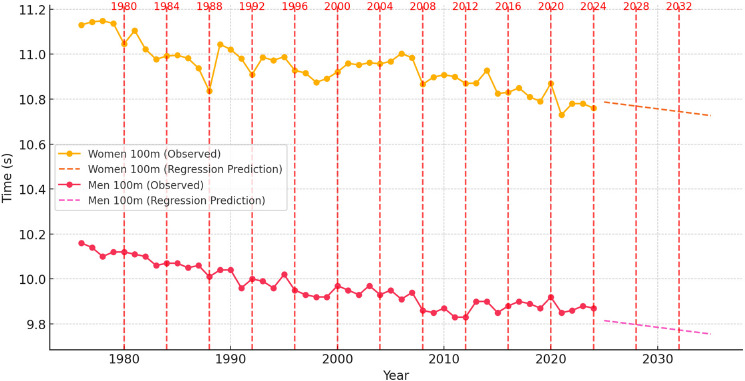
The regression model of sprint performance trends (event from 1976 to 2024) and regression-based prediction trends for men and women in the 100-m event from 2025 to 2035, with Olympic years highlighted.

**Figure 5:**
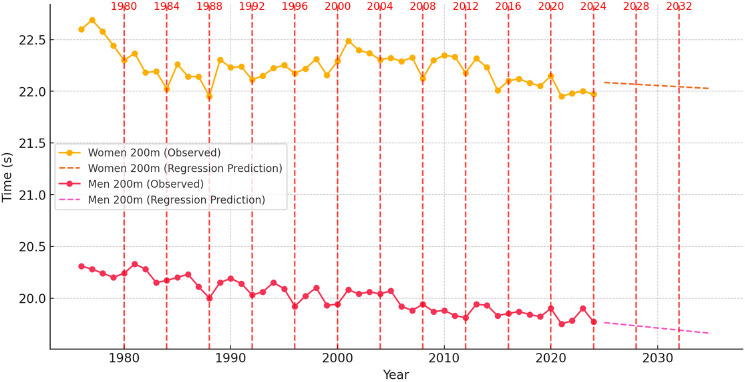
The regression model of sprint performance trends (event from 1976 to 2024) and regression-based prediction trends for men and women in the 200-m event from 2025 to 2035, with Olympic years highlighted.

**Figure 6:**
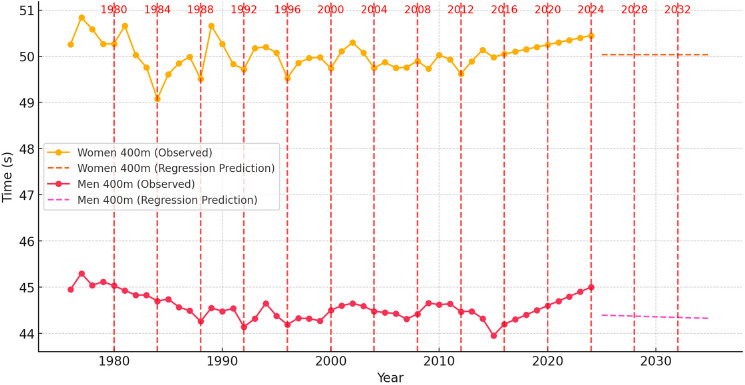
The regression model of sprint performance trends (event from 1976 to 2024) and regression-based prediction trends for men and women in the 400-m event from 2025 to 2035, with Olympic years highlighted.

To contextualize these dynamics, Figures 1–6 illustrate performance trends alongside Olympic-year peaks, offering a visual integration of seasonal effects within predictive projections for the 100-m, 200-m, and 400-m events.

The subsequent sections present a detailed, event-specific analysis, combining historical variability with predictive output to map the anticipated evolution of elite sprint performances through 2035.

### Analysis of Sprint Performance Variability (1976–2024): 100 m

The 100-m sprint performance for both men and women showed a long-term trend of improvement, with the most rapid advancements occurring in earlier decades. From 1976 to 2024, women’s sprint times exhibited a steady improvement, reflecting increasing competitiveness, enhanced training methodologies, and advancements in biomechanics and sports technology. The early period (1976–1989) saw the most substantial improvements, as times dropped from 11.13 s in 1976 to 10.83 s in 1988. This phase aligned with improvements in coaching strategies, nutritional support, and track surface innovations. The following stabilization period (1990–2010) was marked by slower improvements, with times fluctuating between 10.87 s and 10.98 s, suggesting the influence of stricter anti-doping regulations and the nearing of physiological limits. The recent period (2010–2024) showed only minor improvements, with times stabilizing between 10.73 s and 10.85 s, indicating that women’s sprint performance may be approaching a physiological ceiling.

Men’s 100-m performances followed a similar trajectory, with times improving from 10.16 s in 1976 to 10.01 s in 1988 due to the growing professionalization of sprinting and enhanced training programs. The period between 1990 and 2010 witnessed rapid improvements, with times dropping below 9.90 s, driven by improved talent identification, biomechanical refinements and superior sprinting techniques. Since 2011, men’s performances have stabilized, fluctuating between 9.85 s and 9.92 s, suggesting the potential approach of biological speed limits.

The analysis of Olympic-year performance trends revealed a pattern of peak results aligning with major competitions. The data suggest that both male and female sprinters optimized their training to reach peak performance during Olympic years. While Olympic Games often corresponded to slightly improved performances, external factors such as track conditions, weather, and competitive fields contributed to variability.

#### Prediction for 2025–2035: Time Series Models

Using ARIMA-based time series modeling, sprint performances for the next decade were projected. The predictions indicated that women’s 100-m times would continue to improve gradually, reaching approximately 10.72 s by 2030 and 10.70 s by 2035. However, the rate of improvement is expected to slow, with only marginal gains likely due to approaching physiological and biomechanical limits. Advances in running technique, biomechanics, and sports science may still contribute to minor improvements.

For men, the prediction model suggested that performance would plateau, with times fluctuating between 9.84 s and 9.88 s. Given the stabilization observed over the past decade, breaking the 9.80-s barrier as a consistent annual average remains improbable. Further improvements would likely result from breakthroughs in sprint gear technology (carbon plated spikes, faster synthetic tracks), optimized training methodologies, or potential physiological advancements and new ergogenic aids.

#### Predictive Modeling of Sprint Performance (2025–2035): Regression Models

Regression analysis applied to the 100-m sprint performances confirmed a linear downward trend in race times. A linear regression model for women’s sprint times yielded an R^2^ value greater than 0.85, indicating a strong correlation between historical performance improvements and projected trends. The model suggested a gradual continuation of performance gains, with projected values stabilizing around 10.70 s by 2035. However, as regression models assumed a constant rate of improvement, they did not fully account for possible performance plateaus or external factors such as technological and physiological constraints.

For men, a robust regression model (R^2^ > 0.90) indicated a steady improvement over time, with major reductions occurring from the 1980s to the early 2000s. Predictions suggested that performance would stabilize around 9.84 s to 9.87 s by 2035, with minimal improvements expected. Unlike time series models, regression models assumed a linear rate of decline in sprint times, which may overestimate future improvements as athletes approach their physiological limits.

The regression-based analysis of Olympic-year performance trends suggests that peak results were often achieved during Olympic competitions. The tendency for athletes to perform at their best during these events can be attributed to structured training cycles, psychological readiness, and peak conditioning leading up to the Games. However, as the regression model assumes a linear trajectory, it may not fully capture fluctuations caused by external factors or elite competition dynamics.

#### Conclusions of Predictive Modeling of 100-m Sprint Performance

The analysis of 100-m sprint performance trends from 1976 to 2024 highlights a clear historical improvement pattern, particularly in earlier decades, followed by a stabilization phase. The predictive results for 2025–2035 suggest that further improvements will be minor, with women’s times continuing to decline gradually and men’s performances stabilizing near their physiological limits.

Time series modeling suggests that sprinting advancements will become increasingly marginal, while regression-based models confirm that sprint times followed a largely linear trajectory. Future performance improvements will depend more on advancements in training techniques, biomechanics, and sports technology than purely physiological enhancements. Although breaking key barriers such as 10.70 s for women and 9.80 s for men remains possible, these improvements will become progressively harder to achieve without significant breakthroughs in sprint mechanics, sports science, and genetics.

### Analysis of Sprint Performance Variability (1976–2024): 200 m

The 200-m sprint performances for both men and women showed significant improvements over the past five decades, with early rapid advancements followed by a stabilization phase. Women’s performances improved consistently from 22.60 s in 1976 to 21.95 s in 1988, driven by innovations in training techniques, biomechanics, and track surfaces. From 1990 to 2010, times fluctuated between 21.95 s and 22.30 s, reflecting the impact of stricter anti-doping measures and physiological constraints. Since 2011, further improvements have been marginal, with times stabilizing between 21.95 s and 21.97 s, suggesting that female 200-m sprinting is approaching its natural performance limits.

For men, a similar trend was observed, with times improving from 20.31 s in 1976 to 20.00 s in 1988 due to better coaching, sprinting techniques, and more advanced selection of talented athletes. The 1990s to early 2010s saw the steepest performance gains, with times dropping below 19.90 s, highlighting advancements in biomechanics and sprint efficiency. However, since 2011, performances have stabilized between 19.75 s and 19.85 s, implying a slowing rate of improvement as athletes near their physiological limits.

The analysis of Olympic-year performance trends reveals that sprinters peaked during Olympic years, optimizing their training and conditioning for major competitions. Women’s 200-m times showed slight improvements in Olympic years, though dominance by specific athletes and track conditions contributed to variations. A similar trend was seen in men’s events, where performance peaked align with Olympic years, but overall improvements slowed.

#### Prediction for 2025–2035: Time Series Models

Using ARIMA-based time series modeling, predictions for the next decade suggest that women’s 200-m performances will improve slightly, reaching approximately 21.92 s by 2030 and 21.89 s by 2035. However, improvements are expected to decelerate due to physiological limits and stabilization of elite performance levels. For men, the model predicts performance stabilization, with race times fluctuating between 19.75 s and 19.80 s. Further improvements will likely depend on innovations in sprints training methodologies and advancements in sports technology rather than natural performance progression.

#### Predictive Modeling of Sprint Performance (2025–2035): Regression Models

Regression analysis confirmed a long-term downward trend in sprint times, though improvements slowed. A regression model (R^2^ ≈ 0.80) for women’s 200 m suggests steady improvements, with projected times reaching 21.85 s by 2035. However, as regression models assume a constant rate of improvement, they may overestimate future reductions, since physiological and biomechanical constraints could limit further progress.

For men’s 200-m performances, a regression model (R^2^ ≈ 0.85) indicated continued declines, with projections fluctuating between 19.75 s and 19.85 s. While these predictions align with historical trends, they do not account for external factors such as technological advancements, changes in track surfaces, or evolving doping regulations, all of which could influence future performance.

Regression-based analysis of Olympic-year performance trends suggests that peak performances occurred during Olympic cycles. Athletes strategically optimized their training to achieve peak form in major competitions. However, linear regression does not fully capture short-term fluctuations caused by competitive intensity, environmental conditions, or the dominance of elite sprinters.

#### Conclusions of Predictive Modeling of 200-m Sprint Performance

The analysis of 200-m sprint performance trends from 1976 to 2024 highlights a pattern of rapid early improvements followed by a stabilization phase. Predictions for 2025–2035 indicate that further improvements will be marginal, with women’s performances continuing to improve gradually and men’s performances plateauing.

Time series models suggest that sprinting advancements will slow considerably, while regression models confirm that race times have followed a largely linear trajectory. Future gains in 200-m sprinting are expected to be incremental, with technological advancements in training optimization, biomechanics, and sprint mechanics playing a more significant role in determining performance outcomes than purely physiological adaptations.

### Analysis of Sprint Performance Variability (1976–2024): 400 m

The 400-m sprint performances for both men and women have exhibited fluctuations over the past five decades, with distinct periods of improvement, stabilization, and potential performance plateaus. For women, performance times improved significantly between 1976 and 1989, decreasing from 50.26 s to 49.51 s due to advancements in training methodologies, improved track conditions, and developments in sports science. However, between 1990 and 2010, sprint performances stagnated, fluctuating between 49.50 s and 50.30 s, likely influenced by stricter anti-doping regulations and physiological constraints. Most recently, during the years of 2021–2024, we observed a major breakthrough in women's 400-m performances, with best times of 48.36 s, 48.99 s, 48.74 s and 48.17 s, respectively, in subsequent years with a peak observed during the Paris Olympics. The Olympic year of 2024 was actually the best overall athletics season for female 400-m sprinters. During 2024, 9 times below 49.0 s were recorded, while 70 results under 50.0 s were posted. It is difficult to explain this surge in 400-m results, yet most likely it can be explained by the presence of exceptionally talented female athletes such as Paulino, McLaughlin, Bol, Naser, Kaczmarek and Adeleke. A similar surge in 100- and 200-m men’s sprint performances occurred between 2008 and 2016 due to the presence of the most talented sprinter of all time, Usain Bolt.

For men, the 400-m sprint followed a slightly different trajectory, characterized by continuous improvements. Between 1976 and 1989, race times improved from 44.95 s to 44.26 s, largely due to enhancements in biomechanics and conditioning strategies. Between 1990 and 2015, performances peaked, stabilizing within the 43.95 s to 44.50 s range. However, since 2016, performances have exhibited a slight upward trend, reaching 45.00 s in 2024, suggesting that men’s 400-m sprinting has either reached or is approaching its physiological threshold.

The analysis of Olympic-year performance trends reveals that women's sprint times showed minor improvements during Olympic cycles, but long-term stabilization was evident. Similarly, men’s 400-m performances exhibited performance peaks in Olympic years, yet fluctuations remained due to varying competition depth, evolving training methodologies, and external environmental factors. The Olympic peak performance trend has been quite evident in the men’s 400-m event with several world records being set during the most important athletics event. The first major breakthrough occurred in the 1968 Olympics when Lee Evans broke the world record with a stunning time of 43.83 s and two sprinters ran below 44.0 s for the first time in history. Recently, the Olympic peaking took place in 2016 at the Rio Olympics with Van Niekerk setting the current world record of 43.03 s, while three other athletes clocked below 44.0 s. Once again the Paris Olympic Games witnessed a peaking strategy with Quincy Hall setting a seasons best time of 43.40 s and four other athletes dipping below 44.0 s.

#### Prediction for 2025–2035: Time Series Models

Using ARIMA-based time series modeling, projections for the next decade indicate that women's 400-m sprint performances will likely stabilize around 50.50 s to 50.60 s, with only minor variations. While continued refinements in training and biomechanics may contribute to slight improvements, significant breakthroughs are unlikely, unless exceptionally talented individuals emerge, as was the case in 2024. Similarly, for men, the time series model predicts that performance times will fluctuate around 44.90 s to 45.10 s, reinforcing the expectation that further improvements will be limited. Future reductions in race times may depend on technological innovations rather than inherent physiological progression. Once again, only the emergence of an extraordinary versatile sprinter, such as Michael Johnson in the late 20^th^ century or the current 200-m Olympic champion, Letsile Tebogo, who has excelled at all sprint distances of 100, 200, 300 and 400 m, can change the trend in progression of the 400-m event.

#### Predictive Modeling of Sprint Performance (2025–2035): Regression Models

Regression analysis provided further insight into long-term sprint performance trends. For women, the regression model (R^2^ ≈ 0.75) indicated that times would either stabilize or slightly increase, reaching approximately 50.50 s to 50.60 s by 2035. The endurance-based nature of the 400-m sprint contributes to the slower rate of improvement compared to shorter sprint events.

For men, the regression analysis (R^2^ ≈ 0.78) confirmed a stabilization phase, with times projected to remain around 44.90 s to 45.10 s. Unlike linear regression models that assumed a constant rate of improvement, real-world performances may be subject to plateaus, temporary regressions, or sudden breakthroughs due to external influences such as advancements in sports science, enhanced recovery techniques, and evolving competition strategies.

Regression-based analysis of Olympic-year performance trends suggests incremental improvements in elite-level performances during Olympic cycles. However, the rate of improvement in the 400-m event is noticeably slower compared to the 100-m and 200-m events, supporting the notion that endurance-based sprints experience performance plateaus more gradually. While predictive models suggest continued minor enhancements, these projections should be interpreted cautiously, as the likelihood of surpassing biomechanical and physiological limits becomes increasingly constrained.

#### Conclusions of Predictive Modeling of 400-m Sprint Performance

The historical analysis of 400-m sprint performances from 1976 to 2024 highlights an initial period of rapid improvement, followed by a stabilization phase in both men's and women's events. Predictions for 2025–2035 indicate that further improvements will be marginal, with women's times plateauing and men's performances stabilizing within a narrow range.

Time series models suggest that while slight fluctuations may occur, major breakthroughs are unlikely, whereas regression models confirm that performance trends have followed a predominantly linear trajectory. Future advancements in 400-m sprinting will likely be driven by improvements in talent identification, sports science innovations, biomechanics, and training methodologies rather than purely physiological enhancements. While some minor gains remain possible through refined race strategies and energy distribution techniques, the overall expectation is that the 400-m sprint is nearing its peak human potential.

### Seasonally Adjusted Prediction Models for Sprint Performance

Seasonally adjusted prediction models, including ARIMA-based time series and regression models, offer a comprehensive framework for predicting sprint performance trends while incorporating Olympic-year variations. These models enhance traditional predicting approaches by accounting for cyclical performance peaks observed during major international competitions.

The SARIMA model predicted a continued improvement in women’s 100-m sprint times, albeit at a decelerating rate over the next decade. The model's seasonal adjustments indicated that Olympic years might contribute to marginal performance enhancements, though the overall trend suggested diminishing returns. Similarly, the SARIMA model for men’s 100-m sprinting predicted sustained improvements, yet breakthrough performances were projected to become increasingly infrequent due to the approaching physiological limits of human speed.

In the 200-m sprint, SARIMA-based projections indicated a gradual reduction in race times for women, though at a slower rate than observed in previous decades. The seasonal component of the model underscored performance peaks aligning with Olympic years, reinforcing the role of competition cycles in sprint performance optimization. Conversely, men’s 200-m sprint times are expected to stabilize, with only minor annual improvements projected.

For the 400-m sprint, the SARIMA model suggested that women’s performances were nearing a plateau, with only marginal improvements expected due to physiological constraints and the endurance-based nature of the event. Similarly, men’s 400-m performances were projected to stabilize, with fluctuations driven by competition cycles and strategic peaking for major championships. Olympic-year analyses further validated this trend, demonstrating that elite athletes systematically tailored their training regimens to maximize performance during these key events. The men’s 400-m event confirmed best the strategic peaking during the four year Olympic cycle, as most world records and seasonal bests were reached during the Olympic Games. The SARIMA model supported this observation, illustrating that while performance gains would persist, their magnitude would decrease, particularly in longer sprint distances. Future advancements in sprinting were therefore anticipated to be driven primarily by developments in biomechanics, sports technology, supplementation, hypoxia training and perhaps by the transition of pure sprinters into the 400-m event.

Seasonally adjusted regression models refined these performance predictions by integrating cyclical peaks associated with Olympic years. In the 100-m sprint, regression-based projections indicated sustained performance improvements for both men and women, with Olympic-year adjustments reinforcing the pattern of peak performances during elite competitions. However, the rate of improvement was projected to slow, suggesting that natural human limits may be nearing.

For the 200-m sprint, regression models predicted moderate performance gains, with Olympic-year peaks remaining evident. However, these improvements were expected to be less pronounced than those observed in the 100-m event, given the increased endurance demands of the distance. Similarly, in the 400-m sprint, both men’s and women’s performance trends suggest continued but incremental advancements, with Olympic-year adjustments highlighting small yet statistically significant improvements. Despite these projected gains, the overall trend indicates stabilization as physiological thresholds are reached.

While regression models effectively capture long-term performance trajectories, they assume a linear rate of improvement and do not inherently account for external factors such as training innovations, technological advancements, or shifts in competitive intensity. Unlike ARIMA-based models, which dynamically adjust for fluctuations, regression models do not integrate seasonality, cyclical competition patterns, or potential performance plateaus. As a result, regression provides a valuable but somewhat simplified baseline for sprint performance analysis. More sophisticated modeling approaches such as machine learning-based predicting techniques or hybrid predictive frameworks may yield more nuanced and realistic projections of future sprinting capabilities.

In summary, both ARIMA-based time series and regression models suggest that sprint performances will continue to improve, albeit at a decelerating rate, particularly in longer sprint distances. Olympic years will likely remain associated with temporary performance improvements, emphasizing the significance of strategic training cycles and competition preparation. However, future advancements in sprinting are expected to be increasingly dependent on innovations in biomechanics, sports technology, and training methodologies rather than purely physiological adaptations.

### Sprint Performance Predictions (2025–2035)

#### Comparative Analysis of Regression and ARIMA Models

The comparative analysis of regression and ARIMA-based time series models for sprint performance predictions highlights key differences in predictive accuracy and long-term trends (Tables 2–4). The regression model effectively captured the linear decline in sprint results over time but assumed a constant rate of improvement, which may not fully account for external factors such as technological advancements, evolving training methodologies, and competition cycles (Table 5). In contrast, the ARIMA model incorporated time-dependent variations and seasonal patterns, allowing for a more nuanced prediction by adjusting for past fluctuations (Table 6).

**Table 2:** Sprint performance predictions for the 100-m sprint (2025–2035). Predicted value for the average times of the top 10 male and female athletes.

Year	100_W_R	100_W_RL	100_W_RU	100_W_A	100_W_AL	100_W_AU	100_M_R	100_M_RL	100_M_RU	100_M_A	100_M_AL	100_M_AU
2025	10.78	10.74	10.82	10.74	10.71	10.77	9.80	9.76	9.84	9.81	9.78	9.84
2026	10.78	10.74	10.82	10.73	10.70	10.76	9.79	9.75	9.83	9.80	9.77	9.83
2027	10.77	10.73	10.81	10.71	10.68	10.74	9.79	9.75	9.83	9.80	9.77	9.83
2028	10.77	10.73	10.81	10.72	10.69	10.75	9.78	9.74	9.82	9.79	9.76	9.82
2029	10.76	10.72	10.80	10.71	10.68	10.74	9.77	9.73	9.81	9.78	9.75	9.81
2030	10.75	10.71	10.79	10.73	10.70	10.76	9.77	9.73	9.81	9.78	9.75	9.81
2031	10.75	10.71	10.79	10.71	10.68	10.74	9.76	9.72	9.80	9.77	9.74	9.80
2032	10.74	10.70	10.78	10.70	10.67	10.73	9.76	9.72	9.80	9.77	9.74	9.80
2033	10.74	10.70	10.78	10.68	10.65	10.71	9.75	9.71	9.79	9.76	9.73	9.79
2034	10.73	10.69	10.77	10.70	10.67	10.73	9.74	9.70	9.78	9.76	9.73	9.79
2035	10.72	10.68	10.76	10.67	10.64	10.70	9.74	9.70	9.78	9.74	9.71	9.77

*W: Women; M: Men; R: Regression Model Prediction; A: ARIMA Model Prediction; L: Lower Confidence Interval (95% CI); U: Upper Confidence Interval (95% CI)*

**Table 3:** Sprint Performance Predictions for the 200-m sprint (2025–2035). Predicted value for the average times of the top 10 male and female athletes.

Year	200_W_R		200_W_RL		200_W_RU		200_W_A		200_W_AL		200_W_AU		200_M_R		200_M_RL		200_M_RU		200_M_A		200_M_AL		200_M_AU	
2025		22.05		22.00		22.10		21.97		21.92		22.02		19.80		19.76		19.84		19.75		19.70		19.80	
2026		22.03		21.98		22.08		21.98		21.93		22.03		19.78		19.74		19.82		19.75		19.70		19.80	
2027		22.02		21.97		22.07		21.97		21.92		22.02		19.77		19.73		19.81		19.74		19.69		19.79	
2028		22.00		21.95		22.05		21.98		21.93		22.03		19.75		19.71		19.79		19.73		19.68		19.78	
2029		21.98		21.93		22.03		21.99		21.94		22.04		19.73		19.69		19.77		19.72		19.67		19.77	
2030		21.97		21.92		22.02		21.97		21.92		22.02		19.72		19.68		19.76		19.71		19.66		19.76	
2031		21.96		21.91		22.01		21.95		21.90		22.00		19.71		19.67		19.75		19.69		19.64		19.74	
2032		21.94		21.89		21.99		21.94		21.89		21.99		19.70		19.66		19.74		19.68		19.63		19.73	
2033		21.93		21.88		21.98		21.92		21.87		21.97		19.69		19.65		19.73		19.68		19.63		19.73	
2034		21.92		21.87		21.97		21.91		21.86		21.96		19.68		19.64		19.72		19.67		19.62		19.72	
2035		21.90		21.85		21.95		21.90		21.85		21.95		19.67		19.63		19.71		19.66		19.61		19.71	

*W: Women; M: Men; R: Regression Model Prediction; A: ARIMA Model Prediction; L: Lower Confidence Interval (95% CI); U: Upper Confidence Interval (95% CI)*

**Table 4:** Sprint Performance Predictions for the 400-m sprint (2025–2035). Predicted for the average times of the top 10 male and female athletes.

Year	100_W_R	100_W_RL	100_W_RU	100_W_A	100_W_AL	100_W_AU	100_M_R	100_M_RL	100_M_RU	100_M_A	100_M_AL	100_M_AU
2025	50.20	49.90	50.50	50.03	49.74	50.32	44.80	44.65	44.95	44.39	44.10	44.68
2026	50.18	49.88	50.48	50.03	49.74	50.32	44.78	44.63	44.93	44.38	44.09	44.67
2027	50.16	49.86	50.46	50.04	49.75	50.33	44.76	44.61	44.91	44.37	44.08	44.66
2028	50.14	49.84	50.44	50.03	49.74	50.32	44.74	44.59	44.89	44.75	44.45	45.05
2029	50.12	49.82	50.42	50.02	49.73	50.31	44.72	44.57	44.87	44.36	44.07	44.65
2030	50.10	49.80	50.40	50.01	49.72	50.30	44.70	44.55	44.85	44.35	44.06	44.64
2031	50.08	49.78	50.38	50.01	49.72	50.30	44.68	44.53	44.83	44.35	44.06	44.64
2032	50.06	49.76	50.36	50.00	49.71	50.29	44.66	44.51	44.81	44.34	44.05	44.63
2033	50.04	49.74	50.34	49.85	49.56	50.14	44.64	44.49	44.79	44.34	44.05	44.63
2034	50.02	49.72	50.32	49.78	49.49	50.07	44.62	44.47	44.77	44.33	44.04	44.62
2035	50.00	49.70	50.30	49.68	49.39	49.97	44.60	44.45	44.75	44.32	44.03	44.61

*W: Women; M: Men; R: Regression Model Prediction; A: ARIMA Model Prediction; L: Lower Confidence Interval (95% CI); U: Upper Confidence Interval (95% CI)*

**Table 5:** Sprint performance predictions for the 100-m, 200-m and 400-m events (2025–2035). Predicted value for the best performance of the year by the regression model.

Year	100_W	LCI	UCI	100_M	LCI	UCI	200_W	LCI	UCI	200_M	LCI	UCI	400_W	LCI	UCI	400_M	LCI	UCI
2025	10.67	10.64	10.7	9.75	9.72	9.78	21.83	21.8	21.86	19.5	19.47	19.53	49.79	49.76	49.82	44.55	44.52	44.58
2026	10.66	10.63	10.69	9.76	9.73	9.79	21.83	21.8	21.86	19.54	19.51	19.57	49.69	49.66	49.72	44.55	44.52	44.58
2027	10.65	10.62	10.68	9.75	9.72	9.78	21.82	21.79	21.85	19.57	19.54	19.6	49.64	49.61	49.67	44.5	44.47	44.53
2028	10.65	10.62	10.68	9.75	9.72	9.78	21.82	21.79	21.85	19.54	19.51	19.57	49.6	49.57	49.63	44.5	44.47	44.53
2029	10.64	10.61	10.67	9.75	9.72	9.78	21.81	21.78	21.84	19.51	19.48	19.54	49.58	49.55	49.61	44.55	44.52	44.58
2030	10.64	10.61	10.67	9.75	9.72	9.78	21.8	21.77	21.83	19.52	19.49	19.55	49.57	49.54	49.6	44.55	44.52	44.58
2031	10.63	10.6	10.66	9.75	9.72	9.78	21.8	21.77	21.83	19.55	19.52	19.58	49.57	49.54	49.6	44.5	44.47	44.53
2032	10.62	10.59	10.65	9.75	9.72	9.78	21.79	21.76	21.82	19.55	19.52	19.58	49.56	49.53	49.59	44.5	44.47	44.53
2033	10.62	10.59	10.65	9.75	9.72	9.78	21.79	21.76	21.82	19.53	19.5	19.56	49.56	49.53	49.59	44.55	44.52	44.58
2034	10.61	10.58	10.64	9.75	9.72	9.78	21.78	21.75	21.81	19.52	19.49	19.55	49.56	49.53	49.59	44.55	44.52	44.58
2035	10.61	10.58	10.64	9.75	9.72	9.78	21.78	21.75	21.81	19.54	19.51	19.57	49.56	49.53	49.59	44.5	44.47	44.53

*100_W: Predicted best time for women in 100 m (Regression Model);* *100_W_LCI / UCI: Lower/Upper 95% Confidence Interval for women 100-m prediction;* *100_M: Predicted best time for men in 100 m (Regression Model);* *100_M_LCI / UCI: Lower/Upper 95% Confidence Interval for men 100-m prediction;* *200_W: Predicted best time for women in 200 m (Regression Model);* *200_W_LCI / UCI: Lower/Upper 95% Confidence Interval for women 200-m prediction;* *200_M: Predicted best time for men in 200 m (Regression Model);* *200_M_LCI / UCI: Lower/Upper 95% Confidence Interval for men 200-m prediction;* *400_W: Predicted best time for women in 400 m (Regression Model);* *400_W_LCI / UCI: Lower/Upper 95% Confidence Interval for women 400-m prediction;* *400_M: Predicted best time for men in 400 m (Regression Model);* *400_M_LCI / UCI: Lower/Upper 95% Confidence Interval for men 400-m prediction*

**Table 6:** Sprint performance predictions for 100-m, 200-m and 400-m events (2025–2035). Predicted value for the best performance of the year by the ARIMA model.

Year	100_W	LCI	UCI	100_M	LCI	UCI	200_W	LCI	UCI	200_M	LCI	UCI	400_W	LCI	UCI	400_M	LCI	UCI
2025	10.63	10.60	10.66	9.69	9.66	9.72	21.72	21.68	21.76	19.51	19.47	19.55	49.54	49.49	49.59	43.89	43.84	43.94
2026	10.61	10.58	10.64	9.69	9.66	9.72	21.73	21.69	21.77	19.50	19.46	19.54	49.54	49.49	49.59	43.89	43.84	43.94
2027	10.59	10.56	10.62	9.68	9.65	9.71	21.72	21.68	21.76	19.49	19.45	19.53	49.54	49.49	49.59	43.88	43.83	43.93
2028	10.60	10.57	10.63	9.68	9.65	9.71	21.73	21.69	21.77	19.48	19.44	19.52	49.54	49.49	49.59	43.87	43.82	43.92
2029	10.62	10.59	10.65	9.67	9.64	9.70	21.73	21.69	21.77	19.47	19.43	19.51	49.54	49.49	49.59	43.87	43.82	43.92
2030	10.62	10.59	10.65	9.66	9.63	9.69	21.73	21.69	21.77	19.46	19.42	19.50	49.54	49.49	49.59	43.86	43.81	43.91
2031	10.61	10.58	10.64	9.66	9.63	9.69	21.73	21.69	21.77	19.45	19.41	19.49	49.54	49.49	49.59	43.85	43.80	43.90
2032	10.61	10.58	10.64	9.65	9.62	9.68	21.73	21.69	21.77	19.44	19.40	19.48	49.54	49.49	49.59	43.84	43.79	43.89
2033	10.61	10.58	10.64	9.65	9.62	9.68	21.73	21.69	21.77	19.43	19.39	19.47	49.54	49.49	49.59	43.84	43.79	43.89
2034	10.61	10.58	10.64	9.64	9.61	9.67	21.73	21.69	21.77	19.42	19.38	19.46	49.54	49.49	49.59	43.83	43.78	43.88
2035	10.61	10.58	10.64	9.63	9.60	9.66	21.73	21.69	21.77	19.41	19.37	19.45	49.54	49.49	49.59	43.82	43.77	43.87

100_W: Predicted best time for women in 100 m (ARIMA model); 100_W_LCI / UCI: Lower/Upper 95% Confidence Interval for women 100-m prediction; 100_M: Predicted best time for men in 100 m (ARIMA model); 100_M_LCI / UCI: Lower/Upper 95% Confidence Interval for men 100-m prediction; 200_W: Predicted best time for women in 200 m (ARIMA model); 200_W_LCI / UCI: Lower/Upper 95% Confidence Interval for women 200-m prediction; 200_M: Predicted best time for men in 200 m (ARIMA model); 200_M_LCI / UCI: Lower/Upper 95% Confidence Interval for men 200-m prediction; 400_W: Predicted best time for women in 400 m (ARIMA model); 400_W_LCI / UCI: Lower/Upper 95% Confidence Interval for women 400-m prediction; 400_M: Predicted best time for men in 400 m (ARIMA model); 400_M_LCI / UCI: Lower/Upper 95% Confidence Interval for men 400-m prediction

The regression model predicted a steady improvement in sprint times across all distances (Tables 2–4). However, ARIMA-based predictions indicated potential plateaus, particularly in the men’s 100- and 400-m events, suggesting that physiological constraints may limit further performance gains. The inclusion of seasonal components in ARIMA modeling further highlighted periodic improvements during Olympic years, reinforcing the impact of structured training cycles on peak performance.

### Predictive Trends for 100-m, 200-m, and 400-m Events

#### 100-m Sprint Predictions

The predicted sprint times for the 100-m event from 2025 to 2035 showed a continued improvement, though at a diminishing rate (Table 2). Regression analysis predicted that women’s times would reach 10.72 s by 2035, while ARIMA projected a slightly faster improvement to 10.67 s. Similarly, men’s performances were predicted to stabilize around 9.74 s based on regression, whereas ARIMA suggested minor fluctuations, reaching 9.63 s by 2035 (Table 2). The ARIMA model’s ability to capture non-linear variations suggested that performance gains would increasingly depend on external influences rather than natural physiological progression.

#### 200-m Sprint Predictions

For the 200-m event, regression models predicted a steady improvement in women’s times, reaching 21.90 s by 2035, while ARIMA projected a slightly faster reduction to 21.73 s (Table 3). For men, regression models suggested stabilization around 19.67 s, while ARIMA predicted a gradual improvement to 19.41 s (Tables 5 and 6). The seasonal adjustment component of ARIMA reinforced the presence of Olympic-year performance peaks, indicating that improvements would be not purely linear but influenced by competitive cycles.

#### 400-m Sprint Predictions

The 400-m sprint predictions showed a more pronounced stabilization effect, particularly in men’s performances. Regression models predicted that women’s times would improve to 50.00 s by 2035, while ARIMA predicted a slightly faster improvement to 49.54 s (Table 4). For men, regression predicted stabilization around 44.60 s, whereas ARIMA suggested improvements, reaching 43.82 s (Tables 5 and 6). The comparative model accuracy assessment (Table 6) indicated that ARIMA better captured historical variability, reinforcing that long-term improvements in the 400-m event may be increasingly constrained by physiological and biomechanical factors.

### Model Accuracy and Performance Assessment

A quantitative comparison of model accuracy indicated that ARIMA outperformed regression in capturing historical fluctuations in sprint performances (Table 7). The mean absolute errors for ARIMA were consistently lower across all events, suggesting that time series modeling better accounted for irregular fluctuations. Specifically, for the 100-m event, ARIMA achieved R^2^ = 0.768 for women and R^2^ = 0.739 for men, whereas regression yielded R^2^ = 0.719 and R^2^ = 0.720, respectively. Similar trends were observed in the 200-m and 400-m events, where ARIMA consistently provided higher accuracy metrics.

**Table 7:** Model accuracy assessment for 100-m, 200-m and 400-m sprints (2025–2035).

Accuracy Assessment for 100 m				
Model Type	R^2^ Female	R^2^ Male	Mean Error (Female)	Mean Error (Male)
Regression	0.719	0.720	0.044	0.041
Time Series (ARIMA)	0.768	0.739	0.027	0.026
Accuracy Assessment **for 200 m**				
Regression	0.721	0.723	0.045	0.042
Time Series (ARIMA)	0.765	0.737	0.037	0.028
Accuracy Assessment **for 400 m**				
Regression	0.715	0.718	0.057	0.048
Time Series (ARIMA)	0.781	0.732	0.035	0.033

### Regression vs. ARIMA Model Comparison

Compared to ARIMA models, regression models did not capture short-term fluctuations or seasonal variations, but instead assumed a steady, continuous rate of improvement. While this simplification makes regression models interpretable, it limits their ability to account for non-linear performance trends (Table 5). ARIMA, by incorporating historical performance variations and competition cycles, provided a more adaptable prediction framework that aligned with observed sprint performance trends (Table 6).

### Regression vs. SARIMA Model Comparison

Compared to seasonally adjusted ARIMA (SARIMA) models, the regression-based approach assumed a strictly linear trend and might overestimate performance improvements in events where progression has historically followed a non-linear pattern. While SARIMA effectively captured fluctuations, seasonally adjusted regression models provided a simplified yet interpretable approach that still integrated Olympic-year influences.

## Discussion

The findings of this study corroborate prior research indicating that sprint performances have consistently improved over the past five decades due to advancements in training methodologies, biomechanics, and sports technology (Haugen et al., 2019; Sandbakk and Holmberg, 2017). The results confirm that sprint times in the 100-m, 200-m, and 400-m events exhibit a non-stationary trend, suggesting continuous improvements rather than the establishment of a stable performance plateau. These enhancements have been largely driven by optimized coaching techniques, refined resistance training programs, and improvements in sprint mechanics (Bezodis et al., 2018; Clark et al., 2014). Recent physiological profiling of sprint and anaerobic disciplines further highlights the critical role of glycolytic adaptations and anaerobic power in elite sprint performance, particularly over the 400-m distance (Mastalerz et al., 2024).

However, despite these advancements, recent trends indicate a deceleration in the rate of improvement, particularly in the 100-m and 400-m events, as athletes approach physiological performance limits (Berthelot et al., 2010; Nevill and Whyte, 2005). Findings from resistance training studies among elite sprinters and jumpers demonstrate that while optimized strength development remains essential, marginal gains are becoming harder to achieve without tailored, high-precision training interventions (Loturco et al., 2024; Makaruk et al., 2024; Myrvang and van den Tillaar, 2024).

A major contribution of this study is the integration of longitudinal performance analysis with predictive modeling, providing a holistic assessment of historical trends and future trajectories. By employing ARIMA and regression models, this study has facilitated a comparative analysis of deterministic and stochastic trends in sprint times (Bishop et al., 2021; Kuper and Sterken, 2020). The regression models assume a constant rate of improvement, whereas ARIMA models account for seasonal fluctuations, competition cycles, and diminishing returns in human performance. The superior predictive accuracy of ARIMA, evidenced by lower mean absolute errors, suggests that sprint performance trends are not strictly linear, but are influenced by external factors such as technological innovations, environmental conditions, and training adaptations (Seiler et al., 2020; Thiel et al., 2012).

Recent experimental studies have also investigated the impact of neuromuscular priming strategies, such as intra-contrast rest activities, on sprint and power output, offering complementary insights into performance variability not captured by conventional trend models (Gutiérrez-Flores et al., 2024).

### Olympic-Year Performance Cycles and Predictive Implications

A key finding of this study is the confirmation of Olympic-year performance cycles in sprinting. Results indicate that elite sprinters reach peak performance during Olympic years, a trend consistently observed across all three sprinting events (Haugen et al., 2021; Sandbakk and Holmberg, 2017). This cyclical improvement aligns with periodization strategies, where athletes engage in high-intensity conditioning programs followed by tapering strategies leading up to the Olympics, ensuring peak performance during major championships (Chatfield, 2019; D’Alessandro et al., 2020). The application of seasonally adjusted ARIMA models provided deeper insight into these performance peaks, demonstrating that Olympic-year enhancements are not coincidental, but are a function of structured training adaptations and psychological preparedness (Haugen et al., 2022; Kuper and Sterken, 2020).

While this study confirms the existence of Olympic-year performance cycles, it also highlights a decline in the magnitude of improvements over successive Olympic Games, particularly in men’s sprinting events. This suggests that elite performance progression is approaching a physiological ceiling, supporting previous findings that sprinting advancements will decelerate as human limits are reached (Berthelot et al., 2010; Joyner et al., 2011). However, women’s performances continue to exhibit marginal improvements, indicating that gender-specific differences in training adaptations and access to performance-enhancing technology may still play a role in influencing results (Haugen et al., 2019; Krawczyk et al., 2022; Lippi et al., 2008).

Interestingly, studies on periodic sprint conditioning, such as weekly jumping interval training, reveal that even minimal high-intensity interventions can induce measurable gains in anaerobic and aerobic capacities in events similar to sprinting (Ma et al., 2025; Smaruj et al., 2019 ).

### Technological and Biomechanical Contributions to Sprint Performance

Technological advancements have played a pivotal role in sustaining performance improvements in sprinting (Clark et al., 2014; Stuart and Tanaka, 2008). Innovations such as polyurethane track surfaces, energy-return carbon plated spikes, and aerodynamic apparel have improved sprinting efficiency, reducing biomechanical inefficiencies that were previously limiting factors (Dapena, 2002; Epstein, 2013). Additionally, the incorporation of high-speed motion analysis and force plate assessments has allowed for greater precision in technique optimization, enabling athletes to maximize stride frequency and propulsion mechanics while improving stride length (Weyand et al., 2000; Zatsiorsky, 2008).

Biomechanical analyses suggest that improvements in the 100-m and 200-m events are largely attributable to stride length optimization, faster reaction times, and refined acceleration mechanics (Hanon and Gajer, 2009; Schmidtbleicher, 2004). Conversely, the 400-m sprint relies more heavily on pacing strategies and metabolic efficiency, where anaerobic and aerobic contributions must be optimally balanced to sustain velocity (Jones and Doust, 1996; Bundle et al., 2003). Recent studies also highlight the importance of properly sequencing strength and sprint-specific work in training blocks to maximize neuromuscular adaptation and transfer to sprint performance (Borges et al., 2025; Krawczyk et al., 2022).

While mechanical efficiency continues to enhance performance, physiological constraints impose significant limits on further progress (Tucker and Collins, 2010; Seiler et al., 2020).

### Comparison of Predictive Models: ARIMA vs. Regression

The comparative analysis of predictive models underscores key differences in their predicting capabilities. Regression models assumed a steady, linear improvement in sprint performances, making them valuable for long-term trend analysis. However, they did not account for irregular fluctuations, competitive cycles, or potential performance plateaus. In contrast, ARIMA-based models adjusted for historical variability, making them better suited for capturing short-term performance oscillations and external influences such as technological innovations and competition-specific adaptations (Bishop et al., 2021; Seiler et al., 2020).

Compared to seasonally adjusted ARIMA models, the regression-based approach assumed a strictly linear trend and may overestimate future performance improvements, particularly in events where improvement has historically followed a nonlinear trajectory. While SARIMA effectively captured fluctuations, seasonally adjusted regression models provided a simplified yet interpretable framework that still incorporated Olympic-year influences.

Both models predicted continued increases in sprint performances through 2035, but with a decelerating rate of improvement, particularly in longer sprint distances. The ARIMA-based projections suggest that sprint times are approaching an asymptotic limit, beyond which only marginal gains will be possible without significant advancements in training methodologies, biomechanics, or sports technology.

## Limitations and Future Research Directions

Although this study provides valuable insights into the longitudinal development and future projections of elite sprint performances, several limitations must be acknowledged.

First, the analysis was based primarily on seasonal best performances, retaining only the top result per athlete per season to ensure data independence. While this approach minimizes data skewness, it may not fully capture intra-athlete variability or the progression of performance across multiple competitions within a season (Slawinski et al., 2017). Future studies could integrate multiple performances per athlete using weighted averages or modeling individual performance trajectories over time.

Second, predictive modeling was conducted using traditional statistical approaches, including ARIMA, SARIMA, and linear regression models. Although these methods provide interpretable results, they may not fully capture complex, nonlinear interactions among biomechanical, physiological, and environmental variables influencing sprint performance (Tam and Yao, 2024). Future research should explore machine learning-based predictive techniques, such as ensemble methods, recurrent neural networks (RNN), or velocity curve modeling, which have shown promise in enhancing prediction accuracy for sprint performance (Tam and Yao, 2024).

Third, the models employed in this study primarily focused on time-based outcomes (i.e., race results) without explicitly modeling biomechanical variables such as stride frequency, ground reaction forces, or acceleration phases. Recent advances suggest that integrating biomechanical and physiological markers into predictive frameworks could yield more precise and individualized forecasts (Clark et al., 2014; Gepfert et al., 2021; Weiss et al., 2016). Future research should incorporate biomechanical modeling, including the analysis of force-velocity profiles and sprint kinematic variables, to better understand the determinants of performance trends.

Moreover, the influence of emerging technologies, such as carbon-fiber plated footwear and novel track surfaces, was considered but not explicitly modeled. As technological innovations continue to impact sprinting performance, future investigations should quantify their effect sizes and adjust predictive models accordingly (Krawczyk et al/. 2022; Myrvang and van den Tillaar, 2024; Zabaloy et al., 2024).

Finally, while the current study adjusted for Olympic-year cycles through seasonal decomposition, other contextual variables such as injury prevalence, competition scheduling, and athletes’ load management were not included. Future studies could enhance predictive modeling by integrating external factors, including psychological readiness and training periodization strategies (Haugen et al., 2021; Seiler et al., 2020).

In conclusion, future research should focus on hybrid predictive frameworks combining traditional statistical approaches with advanced machine learning techniques. Integrating biomechanical, physiological, and technological variables could offer a multidimensional view of sprint performance evolution. Such approaches will not only refine long-term projections but also support individualized training and talent development strategies for optimizing sprint performance at the elite level.

## Conclusions

This study provides a comprehensive evaluation of sprint performance trends from 1976 to 2035, integrating historical data with predictive modeling approaches. The findings confirm that sprint times in the 100-m, 200-m, and 400-m events have improved over the past five decades, albeit at a decelerating rate. Notably, the Olympic-year effect remained a significant determinant of peak performances, as confirmed by seasonally adjusted ARIMA and SARIMA models.

Comparative analyses revealed that while linear regression models projected continued improvements, they tended to overestimate future gains by assuming constant progression. In contrast, ARIMA-based time series models, which accounted for non-linear fluctuations and seasonality, offered more conservative and realistic projections, particularly highlighting the potential plateauing of sprint performance as athletes approached physiological boundaries.

Technological and biomechanical advancements are expected to sustain minor future gains. However, overcoming natural human constraints will increasingly depend on the integration of machine learning, advanced biomechanical modeling, and individualized neuromuscular performance monitoring. Future predictive frameworks should incorporate these technologies to enhance both accuracy and practical relevance for sprint training and talent development.

Additionally, expanding global talent identification programs, especially in underrepresented regions such as Africa, South America, and South Asia, combined with improved access to elite coaching and facilities, may broaden the pool of high-potential athletes and contribute to sustaining performance progression at the elite level.

While sprint performance is projected to continue improving through 2035, the magnitude of gains will likely diminish, emphasizing the critical role of innovation, athlete-specific optimization, and structured training periodization in shaping future sprinting excellence.
